# Mitogenomes reveal the timing and distribution of divergence events among trans-Beringian birds

**DOI:** 10.7717/peerj.20675

**Published:** 2026-02-02

**Authors:** Keiler A. Collier, Travis C. Glenn, Naoki Takebayashi, Michael J. Hickerson, Kevin Winker

**Affiliations:** 1 University of Alaska Museum, University of Alaska–Fairbanks, Fairbanks, AK, United States of America; 2 Department of Biology and Wildlife, University of Alaska–Fairbanks, Fairbanks, AK, United States of America; 3 Reneco International Wildlife Consultants, Abu Dhabi, Abu Dhabi Emirate, United Arab Emirates; 4 Department of Environmental Health Science, University of Georgia, Athens, GA, United States of America; 5 Institute of Arctic Biology, University of Alaska–Fairbanks, Fairbanks, AK, United States of America; 6 Department of Biology, City University of New York (CUNY) System, New York City, NY, United States of America; 7 CUNY Graduate Center, New York, NY, United States of America; 8 Division of Invertebrate Zoology, The American Museum of Natural History, New York, NY, United States of America

**Keywords:** Beringia, Birds, Mitogenome, Divergence, Speciation, Glaciation

## Abstract

Glacial cycles operating across Beringia have repeatedly exposed large swathes of the Bering Land Bridge, intermittently isolating and reuniting North American and Eurasian taxa. In high-latitude birds, these cycles are hypothesized to have been important in driving divergence and speciation. These repeated events have resulted in multiple trans-Beringian avian sister populations of varying degrees of taxonomic depth distributed across modern Beringia. We asked how these cyclic pulses have affected the temporal distribution and number of overall divergence events across Beringia. We sequenced full mitogenomes at high depth from 39 lineage pairs of varying levels of divergence, totaling 432 individuals of seven orders, 14 families, and 49 species from both Eurasia and North America. We then used a hierarchical approximate Bayesian comparative (hABC) approach to estimate the number and distribution of divergence events between the population pairs, using subsampled datasets. Net nucleotide divergence (*D_A_*) and Jukes-Cantor distance (JC-distance) were also calculated for each pairwise comparison to estimate divergence dates between taxa, using calibrated rates appropriate for shallow avian divergence events. Average divergence times were 200,000 ya for population-level taxa (*n* = 16), 720,000 ya for subspecies (*n* = 12), and 1 Mya for species (*n* = 11), although we consider these dating estimates conservative because of a lack of appropriate calibration for data of this quality. We found eighteen taxon pairs to be significantly differentiated (*p* < 0.05) by *F_ST_* or substantially differentiated by haplotype clade, bounding the number of potential overall divergence events from 1 to 18, and two subsets of the full mitogenomic dataset analyzed in MTML-msBayes strongly supported simultaneous divergence of all Beringian lineages. However, this finding of simultaneous divergence is biologically unusual given the substantial variation in divergence dates among taxa and might indicate a relatively continuous spread of vicariance events, which is difficult to distinguish from a single, simultaneous vicariance event.

## Introduction

Birds are a speciose clade of vertebrates, with more than 11,000 currently recognized species (Gill, Donsker & Rasmussen, 2025). How this diversity is generated has been a central question in evolutionary biology, and avian speciation is considered to be primarily due to selection, as opposed to, *e.g.*, genetic drift (Price, 2008). Speciation is aided considerably by time spent in isolation, as even relatively modest amounts of gene flow can preclude a diverging population from achieving full reproductive isolation (Price, 2008), and hybridization and backcrossing are widespread upon secondary contact in birds (Grant & Grant, 1992; Ottenburghs et al., 2015). At high latitudes, glacial-interglacial cycles are thought to have had major influences on divergence and speciation (Hewitt, 2000; Pielou, 2008). These cycles caused populations to diverge and then potentially reunite as landscapes and ocean levels changed, and mtDNA sequence studies have shown the importance of Pleistocene cycles on avian divergence (*e.g.*, Klicka & Zink, 1997; Arbogast & Slowinski, 1998; Johnson & Cicero, 2004; Weir & Schluter, 2004; Lovette, 2005). Populations have the opportunity to undergo change when separated, especially if divergent selection is operating. Thus far, we know rather little about how the timing and frequency of speciation events in high-latitude systems have been shaped by these glacial cycles. This information from Beringia would complement studies of avian divergence conducted in lower-latitude systems (*e.g.*, Campbell, Braile & Winker, 2016; Miller et al., 2021) and provide greater insight into the processes of avian speciation on a broader scale.

**Figure 1:**
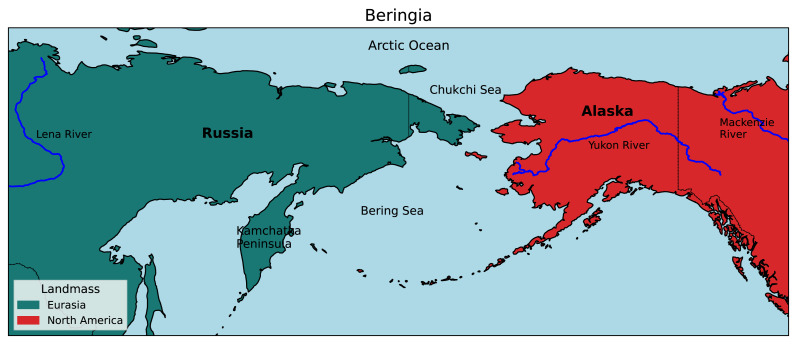
Map of the Beringian region. Beringia is bordered by the Lena and Mackenzie rivers from West to East, and the Arctic Ocean and Kamchatka Peninsula from North to South. (Adapted from the National Park Service, 2020).

Beringia is a high-latitude region bordered longitudinally by the Lena and Mackenzie rivers of Russia and Canada, respectively, and latitudinally by the Chukchi Sea in the north and the tip of the Kamchatka Peninsula in the south (Fig. 1). It has historically been a significant biogeographic link between the Old World and New World, and repeated glacial cycles throughout the Pleistocene (2.6 Mya–10 Kya) have likely caused extensive divergence and speciation by vicariance in a variety of taxa (Lister, 2004; Ikeda & Setoguchi, 2017; McLaughlin et al., 2020).

Beringian birds have yet to be comprehensively investigated in terms of larger-scale divergence assessments, although multiple studies with fewer species have been completed (Zink et al., 1995; Humphries & Winker, 2011; McLaughlin et al., 2020; Spaulding et al., 2022; Oliver Brown, Glenn & Winker, 2025). Collectively, prior research has shown that there is substantial genetic differentiation occurring in the region (including species-level divergences) and that gene flow is common. But a comprehensive analysis of the distribution of divergence events through time, the focus of this study, has not yet been done. Because this region occupies a unique global position at the juncture between Eurasia and North America, many Beringian lineages exist as distinct sister species, subspecies, or populations on both sides. These sister lineages are separated currently by the Bering and Chukchi seas in an intercontinental contact zone and exhibit variable levels of intergradation and divergence; *i.e.,* concordant with population-to-species level expectations (Zink et al., 1995; Humphries & Winker, 2011; Humphries & Winker, 2011; McLaughlin et al., 2020; Spaulding et al., 2022; Oliver Brown, Glenn & Winker, 2025). There are also distinct Beringian avian sister taxa in which one is a regional endemic and the other has a mainland (or other island) sister (Winker et al., 2023).

This study addresses two major questions about the Beringian system. First, how were these events distributed across time? It is possible that avian divergence events were either relatively continuously distributed throughout the Pleistocene or that they were clumped around particularly severe glacial-interglacial cycles. Given the uneven nature of cycle severity (Lisiecki & Raymo, 2005), we predicted that avian divergence events should be clumped in distribution. Second, how common were these divergence events? The exact number of Pleistocene glacial-interglacial cycles is not a settled question, with authorities variously estimating from 11 major cycles to over 100 major and minor cycles (Richmond & Fullerton, 1986; Ruddiman, Raymo & McIntyre, 1986; Lisiecki & Raymo, 2005). Thus, it is unclear how many glacial cycles would have produced divergence events or how clustered individual events might be relative to others. We examined these questions using data from whole mitogenomes and by determining the timing and distribution of divergence events using sequence divergence and both non-Bayesian and hierarchical approximate Bayesian computation (hABC) approaches among 39 pairwise comparisons from a broadly representative set of Beringian birds.

## Materials & Methods

### Taxonomic selection

We selected thirty-nine lineage pairs based on specimen availability and distribution across Beringia. The lineages selected were from six avian orders and 14 families and broadly representative of the Beringian avifauna. For example, our strongest representation is in the three most diverse Beringian orders, with substantial percentages of each of these groups’ available lineages included: Anseriformes 42% (∼13/31), Charadriiformes 17% (∼12/69), and Passeriformes 19% (∼11/59; Table 1 and Winker et al., 2023). For species, we used the framework developed for Beringian birds given in Winker et al. (2023), in which clearly closely related congeners on each side of the Bering and Chukchi seas were considered sisters unless species-level phylogenies (which do not exist for all these genera) showed otherwise. One lineage (*Phylloscopus* spp.) has both subspecific and species-level divergence across Beringia. Lineages show variable biogeographic patterns, including taxa of predominantly Old World (OW; *n* = 202 individuals), predominantly New World (NW; *n* = 230 individuals), and Holarctic distributions, with a limited number of intra-Beringian comparisons, primarily involving endemic taxa. Individuals were categorized as OW or NW based on phenotype except for population-level contrasts, which were based on location. Migrant OW birds often occur in the western Aleutian Islands (NW), and some of these were used to increase sample sizes of OW specimens available (Table S1). Lineages varied in divergence level as reflected in current taxonomy, with 12 species-level, 11 subspecies-level, and 16 population-level divergences (Chesser et al., 2023; Table 1, Table S1).

**Table 1:** Sampling design, taxonomic information, total mitogenome divergences, and divergence date estimates for all lineages in this dataset. Detailed specimen information can be found in Table S1. Taxonomy follows Chesser et al. (2023). OW, Old World; NW, New World. Divergence date estimates are based on ND2 divergences scaled using Lerner et al. (2011) and Arcones, Ponti & Vieites (2011; see Methods).

Taxon	Taxonomic depth	Total samples	OW samples	NW samples	% Full mitogenome JC-distance	Divergence date (Mya)
*Anser albifrons*	Population	8	3	5	0.66	0.327
*Spatula clypeata*	Population	9	5	4	0.35	0.029
*Mareca penelope/M. americana*	Species	16	8	8	1.43	0.496
*Anas platyrhynchos*	Population	12	5	7	0.34	0.239
*Anas acuta*	Population	10	5	5	0.18	0.087
*Anas c. crecca/A. c. carolinensis*	Subspecies	14	7	7	3.19	1.770
*Aythya marila*	Population	10	5	5	1.32	0.629
*Somateria spectabilis*	Population	7	2	5	0.25	0.140
*Somateria mollissima*	Population	10	5	5	0.33	0.157
*Histrionicus histrionicus*	Population	8	6	2	0.05	0.026
*Melanitta americana*	Population	6	2	4	0.12	0.048
*Clangula hyemalis*	Population	13	7	6	0.11	0.031
*Mergus merganser merganser/M. m. americanus*	Subspecies	10	6	4	0.48	0.263
*Lagopus lagopus koreni/L. l. alascensis*	Subspecies	10	5	5	0.30	0.113
*Pluvialis fulva/P. dominica*	Species	8	4	4	3.21	1.563
*Numenius phaeopus variegatus/N. p. hudsonicus*	Subspecies	15	8	7	2.70	1.078
*Arenaria interpres/A. melanocephala*	Species	9	5	4	3.62	1.511
*Calidris alpina arcticola/C. a. pacifica*	Subspecies	8	3	5	2.45	0.896
*Gallinago gallinago/G. delicata*	Species	10	5	5	1.97	1.081
*Tringa brevipes/T. incana*	Species	16	8	8	3.82	1.837
*Tringa nebularia/T. melanoleuca*	Species	10	5	5	1.73	0.700
*Uria aalge*	Population	10	5	5	0.46	0.160
*Uria lomvia*	Population	10	5	5	1.99	1.026
*Larus canus/L. brachyrhynchus*	Species	10	5	5	0.89	0.407
*Larus argentatus vegae/L. a. smithsonianus*	Subspecies	10	5	5	0.41	0.154
*Larus hyperboreus*	Population	10	5	5	0.32	0.140
*Gavia stellata*	Population	10	5	5	0.29	0.000
*Picoides tridactylus/P. fasciatus*	Species	8	3	5	2.89	0.868
*Pica pica/P. hudsonia*	Species	15	8	7	3.00	1.348
*Corvus corax*	Population	17	7	10	0.56	0.139
*Phylloscopus examinandus/P. borealis*	Species	10	5	5	2.74	0.814
*Phylloscopus borealis borealis/P. b. kennicotti*	Subspecies	15	5	10	2.53	0.741
*Cyanecula svecica*	Population	16	8	8	0.16	0.048
*Motacilla tschutschensis tschutschensis/M. t. simillima*	Subspecies	10	5	5	0.69	0.369
*Anthus rubescens japonicus/A. r. pacificus*	Subspecies	10	5	5	2.70	1.293
*Pinicola enucleator tschutschensis/P. e. flammula*	Subspecies	13	6	7	2.06	0.938
*Leucosticte arctoa/L. tephrocotis*	Species	24	5	19	1.31	0.524
*Calcarius lapponicus coloratus/C. l. alascensis*	Subspecies	7	2	5	2.27	0.638
*Plectrophenax nivalis/P. hyperboreus*	Species	8	4	4	0.43	0.263

### Mitogenome dataset generation

We chose to use mtDNA sequence data for this study to provide a single-locus comparative framework for this system. This framework enables direct contrasts with historic data, the use of good published divergence calibrations, and a computationally tractable dataset for our multi-lineage comparative focus. We have studied some of these same taxa using ultraconserved elements (UCEs) and have found the UCE approach is unlikely (at this time) to provide robust answers to our focal questions (*e.g.*, Winker, Glenn & Faircloth, 2018; McLaughlin et al., 2020; Spaulding et al., 2022; Oliver Brown, Glenn & Winker, 2025). Here we focus only on the mitogenome. Most taxonomic pairs had a minimum of five individuals of each population (or subspecies or species) per pair. We extracted DNA from high-quality frozen muscle tissues from the collections of the University of Alaska Museum and University of Washington Burke Museum (Table S1) using a DNeasy Tissue Kit, following the manufacturer’s protocol (Qiagen, Valencia, California, USA). Associated voucher specimens are archived at these institutions. We then prepared dual-indexed DNA libraries for each sample using the methods in Glenn et al. (2017), and calibrated them using a Qubit fluorimeter (Invitrogen Inc., Carlsbad, CA, USA). For each lineage in which a pairwise comparison was being made, libraries for all associated specimens were combined into an equimolar 500 ng pool, which was then enriched for ultraconserved element (UCE) loci following Winker, Glenn & Faircloth (2018). Fragment size distributions of each lineage-specific pool were quantified using a Bioanalyzer (Agilent Inc., Santa Clara, CA, USA), and pools were amplified and quantified with qPCR using a commercial kit (Kapa Biosystems, Wilmington, MA, USA). Pools were then combined at equimolar ratios and sequenced on one lane of a paired-end 150 bp (PE150) Illumina HiSeq 2500 (Illumina Inc., San Diego, CA, USA; UCLA Neuroscience Genomics Core). This procedure produces large amounts of mitogenomic data as a by-product, and that is our focus here.

We used Illumiprocessor (v.2.0.6; Faircloth, 2013) with all raw fastq files to trim low-quality (phred score <30; *i.e.,* <99.9% accuracy) bases and clip adapter contamination. For each lineage, we then obtained a full annotated mitochondrial reference sequence from GenBank, from a conspecific if available. If not, we used a reference from a closely related species, typically a congener (Table S2). Phyluce (v.1.5.0; Faircloth, 2016) and several of its dependencies were used to call SNPs from reads in individual fasta files against these reference mitogenomic sequences, lineage by lineage. We next aligned raw reads for each individual to the reference and converted them to .bam files using bwa-mem (v.0.7.7; Li & Durbin, 2009), SAMtools (v.0.1.19; Li et al., 2009), and PICARD (v.1.106; http://broadinstitute.github.io/picard). We then cleaned all sequences to remove unmapped reads and beyond end-of-reference alignments, tagged them with read groups corresponding to their specimen ID number, and marked PCR and sequencing duplicates, also using PICARD. We then obtained coverage summary statistics by taxon (Tables S3, S4) from the sorted .bam files using SAMtools.

Using the Genome Analysis Toolkit (GATK, v.3.3.0; McKenna et al., 2010), we indexed each .bam file and called high-quality SNPs against the reference following an established population genomic pipeline (Harvey et al., 2016). We then generated individual mtDNA fasta sequences for each study individual, also using GATK.

### Gene isolation and divergence estimates

We first aligned full mitogenomes in MEGA X (Stecher, Tamura & Kumar, 2020) using Muscle (v.3.8.1551; Edgar, 2004) on default settings. Due to downstream computational limitations (see ‘MTML-msbayes analyses’), we then created a data subset by trimming the alignments according to the coordinates of three mitochondrial genes—NADH dehydrogenase 2 (ND2), cytochrome b (cyt*b*), and cytochrome c oxidase 1 (CO1) in the annotated reference sequences, producing alignments for each of these genes.

We then partitioned each aligned dataset into groups of OW and NW individuals based on taxonomic identity or specimen location, as applicable. To visualize specimen relatedness, we created haplotype networks and neighbor-joining (NJ) trees for each taxon. Haplotype networks were generated for the ND2 dataset with the median-joining algorithm in the program Network v.4.6.1.1 (Bandelt, Forster & Röhl, 1999). NJ trees for the ND2 dataset were generated in MEGA X. We then labeled and color-coded these trees in FigTree (v1.4.4; Rambaut, 2018).

We calculated between-group genetic distances using JC-distances with a gamma distribution in MEGA X, to match the model used in Campbell, Braile & Winker (2016) (K Campbell, pers. comm., 2022). We used Arlequin 3.5 (Excoffier & Lischer, 2010) to calculate net nucleotide divergence (*D*_*A*_; Nei, 1983). Both metrics are common in the historic literature and thus enable direct comparisons with prior work.

For a subset of seven taxon pairs for which historic Sanger-sequencing data existed (Humphries & Winker, 2011), we also used MEGA X to calculate nucleotide diversity (*π*, Nei & Li, 1979) values in both the historic Sanger data and the current Illumina data. We used these values to inform comparisons between these datasets.

Finally, to estimate the proportion of variance in divergence attributable to taxonomic depth, we performed a linear regression of taxonomic level (population, subspecies, and species) against full mitogenomic JC-distance, as calculated above.

### Dating divergence events

We estimated times of divergence (*t)* for pairwise comparisons for the JC-distance values of the whole mitogenome and ND2 datasets. For whole-mitogenome divergence date estimates, we used calibrations from Quillfeldt (2017) and Arcones, Ponti & Vieites (2021). For our ND2 divergence rate estimates, we used the calibration for shallow avian divergence events from Lerner et al. (2011). As Lerner et al. (2011) used only passerines to determine their rate, we adjusted this base value using ordinal rates from Arcones, Ponti & Vieites (2021) to the avian orders present in this study using the equation (Lerner_scaled_ = (Arcones_Desired_lineage_/Arcones_Passeriformes_) * Lerner_unscaled_). This enabled us to make direct comparisons among taxa. Other mtDNA calibration approaches might be used (*e.g.*, Nabholz, Lanfear & Fuchs, 2016), and can be applied directly to our pairwise divergence calculations, although as our results suggest, we consider that new, shallow calibrations are needed for data of the sort we generated. We made pairwise estimates of *F*_*ST*_ using DnaSP v.5 (Librado & Rozas, 2009).

### MTML-msBayes analyses

We assessed the simultaneous *versus* non-simultaneous divergence (estimating the variation in divergence times) of all taxon pairs using a hierarchical approximate Bayesian computational (hABC) approach implemented in MTML-msBayes (Huang et al., 2011). hABC is a likelihood-free Bayesian inference framework that uses stochastic simulations and summary statistics to approximate posterior distributions of model parameters, incorporating hierarchical structure of parameters. In this case, a coalescent demographic model is used, with demographic parameters drawn from user-specified priors to simulate patterns of sequence variation. Summary statistics from the simulated datasets are then compared to those from the observed data, and posterior parameter distributions are estimated. MTML-msBayes estimates the number of divergence times across all taxon pairs (Ψ), the mean divergence time in coalescent generations (E(*τ*)), and the dispersion index of the divergence times (Ω =Var(*τ*)/E(*τ*)), defined as the variance-to-mean ratio of divergence times. We used the dispersion index, Ω, to test the hypothesis of simultaneous *versus* nonsimultaneous divergence of all taxon pairs (*i.e.,* whether all taxon pairs diverged at the same time). The degree to which Ω values deviate from 0.0 (tested using Bayes factors) is interpreted as greater support for non-simultaneous divergence. For further details see Huang et al. (2011).

Due to the computational intractability of analyzing the entire mitogenome, datasets were assembled for each lineage by realigning the previously generated full mitogenomes to extract (i) the ND2 gene and (ii) a ∼4,000 bp subsampled dataset comprising the ND2, cyt*b*, and CO1 genes (hereafter three-gene). These markers were selected based on their prior use in the literature. We further restricted the dataset to taxa with clear evidence of divergence, reducing the initial 39 lineages to 19. We considered lineages to show evidence of divergence if they possessed a pairwise mitogenomic *F*_*ST*_ significantly different from 0 (13/19), or if haplotype sharing (*via*, for example, introgression, incomplete lineage sorting, or recent divergence) across well-defined OW/NW clades was suspected to have caused a reduced *F*_*ST*_ (6/19). In the six lineages where the latter was the case (*Gallinago* spp., *Numenius phaeopus*, *Calidris alpina*, *Larus canus*, *Pinicola enucleator*, and *Calcarius lapponicus*), this assessment was made based on ND2 haplotype networks, NJ trees, and prior evidence for putative gene flow in these taxa (Zink et al., 1995; Humphries & Winker, 2011; McLaughlin et al., 2020; Spaulding et al., 2022). The cyt*b* and CO1 genes were tested in separate runs of 3 million replicates under identical parameters to the ND2 run.

Following Humphries & Winker (2011) and Topp et al. (2013), we defined a set of Bayesian priors to include as model bounds. We set the upper bound of migration rate, UpperMig, to 10, allowing simulations to assume a maximum of 10 migration events per generation. We kept UpperRec, denoting the upper bound of recombination rate in the study locus, at 0, as recombination is not expected in the avian mitogenome (Berlin, Smith & Ellegren, 2004). The upper bound of ancestral *θ* (upperAncPopSize), denoting relative ancestral population size relative to current size, was set to 0.5, erring on the side of wider tolerance. Coalescent theory, implemented in msBayes’ hierarchical simulation method, would predict that individuals that contributed ancestry to the current population should be at most one half of the current population. As we do not have estimates of effective Pleistocene population sizes for most avian taxa, we allowed this value to vary as needed. Additionally, the wide variety of ecologically diverse taxa in this study makes it unlikely that a single, uniform effective population size pattern holds true across the dataset (*e.g.*, Maley & Winker, 2010; McLaughlin et al., 2020); thus, an unbounded prior is likely better.

During initial data exploration runs consisting of 500,000 replications, *τ* values were left unbounded. However, after several exploratory runs, all probable *τ* values were consistently concentrated between 0 and ∼0.15. For our final analysis we bounded upper *τ* to 0.2 for all subsequent runs, providing increased coverage within the sampling space. We note that our upper bound prior of migration rate (UpperMig) of 10, allowing simulations to assume a maximum of 10 migration (gene flow) events per generation, reflects a level at which populations are converging (Winker, 2021). We erred on the side of a wider tolerance here because seasonal migration and dispersal vary widely across our lineages.

We performed three million replicates for both datasets. Simple rejection methods (Huang et al., 2011) were used to construct the posterior distributions.

## Results

We generated 432 high-quality mitogenomes from 38 avian lineages, representing 49 species (and 24 subspecies within these) from 14 families and seven orders (Table 1, Table S1), with an overall average coverage of 101x (Tables S3, S4). Pairwise comparisons were made between 12 species, 11 subspecies, and 16 populations (Table 1).

### Divergence levels and timing

Pairwise sequence divergence (JC-distance) between lineages for full mitogenome data ranged from 0.0 to 3.82% across all taxa, with an average of 1.39 ± 1.19% (Table 2). Species pairs had a significantly higher average genetic divergence (2.32 ± 1.09%) than population (0.47 ± 0.51%; *p* < 0.00001), but not subspecies pairs (1.66 ± 1.05; *p* = 0.16). Subspecies and population pair divergences also differed (*p* = 0.0003). ND2 divergence depths were generally higher than full mitogenomes, ranging from 0.0 to 4.96% for all taxa, 2.76 ± 1.42% for species, 1.83 ± 1.17% for subspecies, 0.52 ± 0.71% for populations, and an overall average of 1.60 ± 1.46% (Table 2, Table S5). As with full mitogenome data, ND2 divergence levels were significantly different between species and populations and between populations and subspecies (Table 2, Table S5).

**Table 2:** JC distance summary statistics. Values represent full mitogenome and ND2 datasets and are given as % divergence.

Data type	Taxonomic depth	Average % JC-distance	Std.dev % JC-distance	Min. % JC-distance	Max. % JC-distance
Full mitogenome	All	1.39	1.19	0.05	3.82
Full mitogenome	Species	2.25	1.11	0.43	3.82
Full mitogenome	Subspecies	1.80	1.09	0.30	3.19
Full mitogenome	Population	0.47	0.51	0.05	1.99
ND2	All	1.60	1.46	0.00	4.96
ND2	Species	2.62	1.40	0.76	4.96
ND2	Subspecies	2.06	1.35	0.31	4.39
ND2	Population	0.52	0.71	0.00	2.77

**Figure 2:**
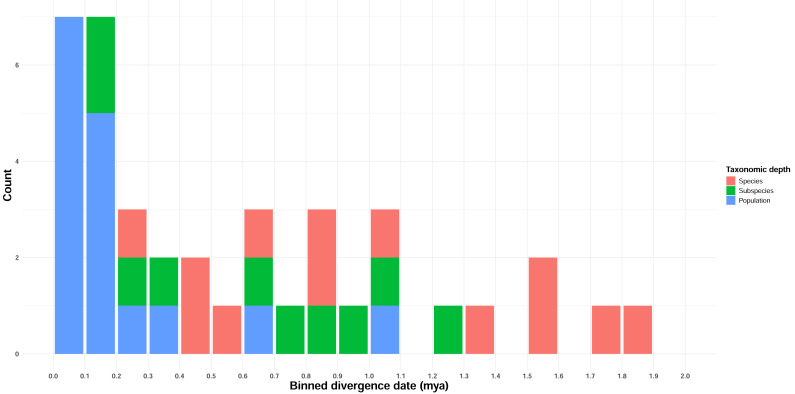
Temporal distribution of divergence date estimates in Beringian birds. Date estimates are derived from ND2 JC-distance estimates, and the frequency distribution is binned by 100 Kyr intervals. Taxonomic depth is indicated by the colors in the figure legend.

Estimated full mitogenome divergence dates between taxon pairs ranged from 69,000 years to 7.96 Mya, with an overall average of 1.95 Mya (Table 3, Table S6). ND2 divergence date estimates, corresponding to the higher divergence rate estimate of Lerner et al. (2011), were lower, ranging from 0 (*Gavia stellata*, which did not have any ND2-specific mutations) to 1.84 Mya, with an overall average of 0.59 Mya (Table 3, Table S6). Divergence estimates increased only loosely with taxonomic depth (Fig. 2, Table 2; Adjusted *R*^2^ = 0.4), although taxonomic level was highly significant in predicting divergence (*p* < 0.001).

**Table 3:** JC-distance-based divergence time summary statistics (Mya). Divergence estimates for full mitogenomes are based on rates in Quillfeldt (2017), and those for ND2 use the rates of Lerner et al. (2011), scaled to non-Passeriformes orders using Arcones, Ponti & Vieites (2021). Values for all pairwise comparisons are given in Table S6.

Data type	Taxonomic depth	Average divergence time	Std.dev divergence time	Min. divergence time	Max. divergence time
Full mitogenome	All	1.95	2.19	0.70	7.96
Full mitogenome	Species	3.61	2.74	0.23	7.96
Full mitogenome	Subspecies	2.02	1.88	0.36	5.63
Full mitogenome	Population	0.76	1.01	0.07	4.15
ND2	All	0.59	0.54	0.00	1.84
ND2	Species	1.00	0.51	0.26	1.84
ND2	Subspecies	0.72	0.50	0.11	1.77
ND2	Population	0.20	0.27	0.00	1.03

Thirteen of 39 taxon pairs were significantly differentiated by pairwise *F*_*ST*_, of which eight are currently classified as species, two as subspecies, and three as populations (Table 4). Of these, all 13 remained significant after false discovery rate adjustment (Benjamini & Hochberg, 1995; Strimmer, 2008) for both the full mitogenome and ND2 datasets.

**Table 4:** Significance tests for population differentiation (*F*_*ST*_). Values of *F*_*ST*_ and tests of significance on the full mitogenome level.

Taxon	Taxonomic depth	Full mitogenome *FST*	Standard deviation	Significance
*Anser albifrons*	Population	−0.092	0.015	0.313
*Spatula clypeata*	Population	−0.092	0.016	0.396
*Mareca penelope/M. americana* ^*^	Species	0.482	0.005	0.035
*Anas platyrhynchos*	Population	0.053	0.012	0.183
*Anas acuta*	Population	−0.026	0.016	0.396
*Anas c. crecca/A. c. carolinensis* ^**^	Subpecies	0.793	0.014	0.002
*Aythya marila* ^*^	Population	0.340	0.004	0.013
*Somateria spectabilis*	Population	0.045	0.014	0.238
*Somateria mollissima*	Population	0.116	0.011	0.169
*Histrionicus histrionicus*	Population	0.090	0.016	0.425
*Melanitta americana*	Population	−0.057	0.012	0.465
*Clangula hyemalis*	Population	−0.015	0.016	0.550
*Mergus merganser merganser/M. m. americanus*	Subspecies	−0.040	0.009	0.897
*Lagopus lagopus koreni/L. l. alascensis*	Subspecies	−0.034	0.010	0.908
*Pluvialis fulva/P. dominica* ^*^	Species	0.967	0.006	0.029
*Numenius phaeopus*	Population	−0.106	0.007	0.941
*Arenaria interpres/A. melanocephala* ^**^	Species	0.886	0.003	0.020
*Calidris alpina arcticola/C. a. pacifica*	Subspecies	−0.010	0.014	0.340
*Gallinago gallinago/G. delicata*	Species	−0.138	0.000	0.999
*Tringa brevipes/T. incana* ^***^	Species	0.958	0.000	0.000
*Tringa nebularia/T. melanoleuca* ^*^	Species	0.401	0.005	0.027
*Uria aalge* ^*^	Population	0.187	0.006	0.045
*Uria lomvia*	Population	0.020	0.013	0.309
*Larus canus/L. brachyrhynchus*	Species	0.137	0.010	0.165
*Larus argentatus vegae/L. a. smithsonianus*	Subspecies	−0.042	0.000	0.999
*Larus hyperboreus*	Population	−0.028	0.016	0.536
*Gavia stellata*	Population	0.043	0.000	0.999
*Picoides tridactylus/P. fasciatus* ^*^	Species	0.627	0.003	0.014
*Pica pica/P. hudsonia* ^***^	Species	0.653	0.000	0.000
*Corvus corax* ^***^	Population	0.122	0.001	0.001
*Phylloscopus examinandus/P. borealis*	Species	0.158	0.013	0.254
*Phylloscopus borealis borealis/P. b. kennicotti*	Subspecies	−0.030	0.018	0.611
*Cyanecula svecica*	Population	−0.022	0.009	0.832
*Motacilla tschutschensis tschutschensis/M. t. simillima*	Subspecies	0.098	0.012	0.188
*Anthus rubescens japonicus/A. r. pacificus* ^**^	Subspecies	0.349	0.010	0.005
*Pinicola enucleator tschutschensis/P. e. flammula*	Subspecies	0.095	0.011	0.107
*Leucosticte arctoa/L. tephrocotis* ^*^	Species	0.463	0.004	0.016
*Calcarius lapponicus coloratus/C. l. alascensis*	Subspecies	0.270	0.009	0.132
*Plectrophenax nivalis/P. hyperboreus*	Species	0.023	0.013	0.524

**Notes.**

* *p* < 0.05.

** *p* < 0.01.

*** *p* < 0.001.

**Figure 3:**
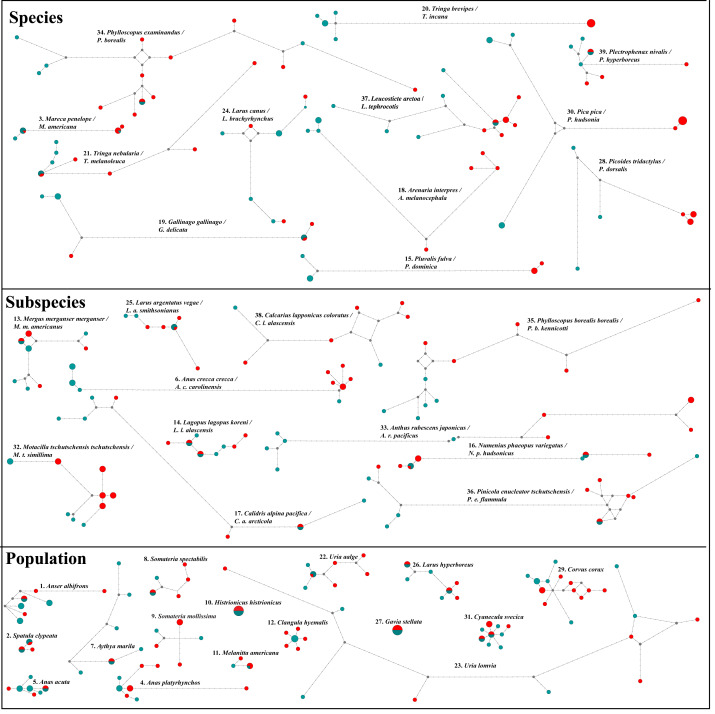
NADH dehydrogenase 2 (ND2) haplotype network diagrams for all taxon pairs. Eurasian taxa/populations are given in aqua and North American taxa/populations in red. Each circle represents a haplotype, and circle size reflects haplotype frequency. Each hatch mark on a line represents a mutation.

Additionally, both datasets in ten pairwise contrasts (*Anas crecca*, *Mareca* spp., *Calidris alpina*, *Gallinago* spp*.*, *Corvus corax*, *Larus canus*, *Numenius phaeopus*, *Anthus rubescens*, *Pinicola enucleator* and *Calcarius lapponicus*) showed strong NW/OW (Fig. 3) clades with varying amounts of haplotype sharing. Prior work in these taxa also showed this pattern (Zink et al., 1995; Humphries & Winker, 2011; McLaughlin et al., 2020; Spaulding et al., 2022; Doniol-Valcroze et al., 2023). However, in all cases, we were unable to distinguish between incomplete lineage sorting and putative gene flow. While distinguishing between incomplete lineage sorting and gene flow is possible with large-scale transposable element data (*e.g.*, Doronina et al., 2015; Suh, Smeds & Ellegren, 2015), these data were not available to us for any of the 39 taxon pairs investigated.

As *F*_*ST*_ is directly affected by gene flow, the ten taxa that showed strong NW/OW haplotype clades were considered to be substantially differentiated and were included in further analyses with the 13 taxa differentiated by *F*_*ST*_. This produced a total subset of 19 taxa, as *Anas crecca*, *Mareca* spp., *Corvus corax*, and *Anthus rubescens* were already significantly differentiated by pairwise *F*_*ST*_ before considering haplotype clades.

### Divergence events

**Figure 4:**
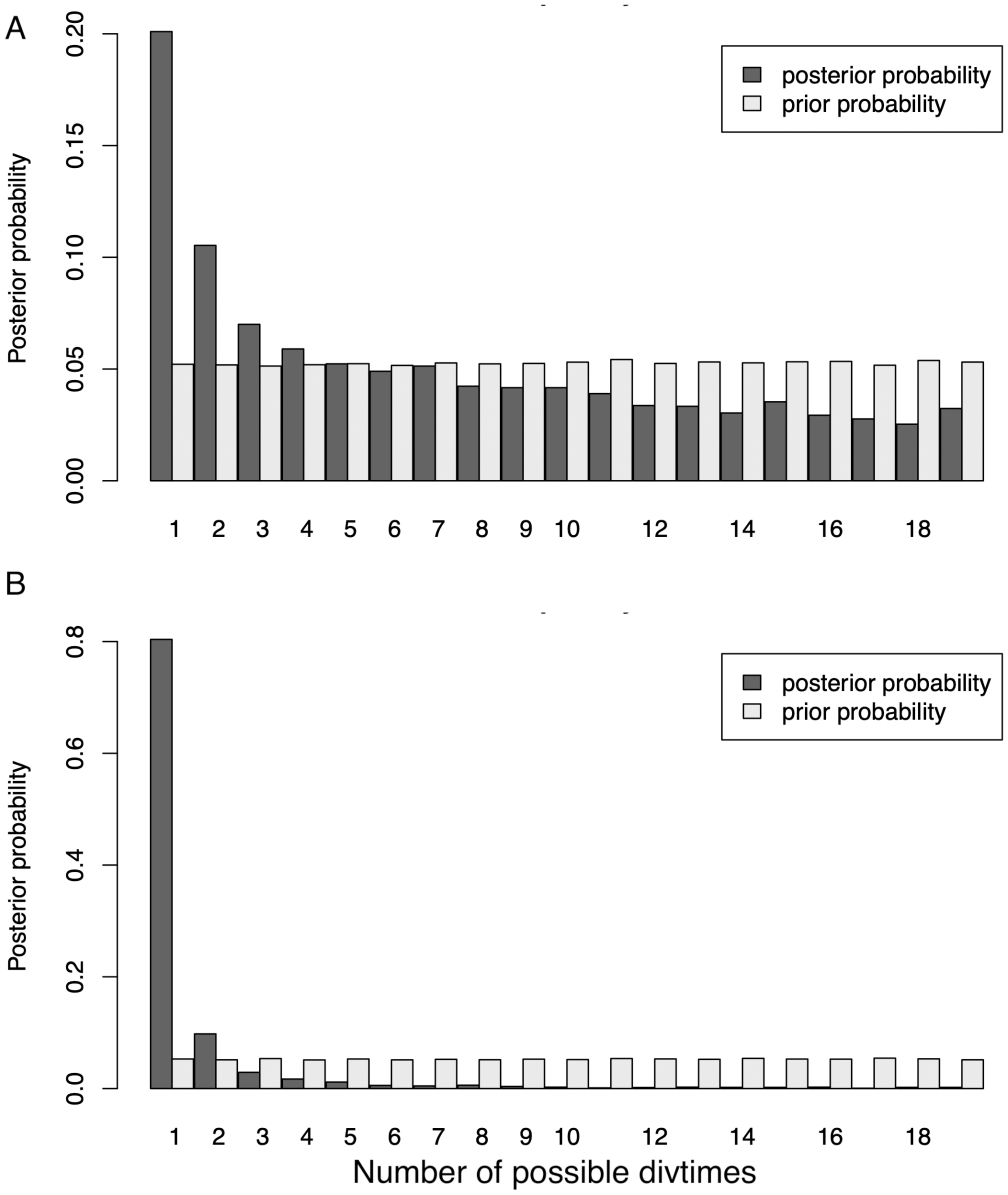
Distribution of Ψ (Estimated number of divergence events) from the Ψ-unconstrained MTML-msBayes analyses of the (A) ND2 and (B) 3-gene datasets. The posterior probability of any given number of divergence events is indicated by the height of the bar, and the sum total of all possible divergence events must equal one. While both the ND2 and 3-gene datasets show one divergence event as the most likely scenario, the larger, 3-gene dataset has substantially more support for a single vicariance event than the ND2 subset.

Our initial ND2 dataset unambiguously showed the strongest posterior probability support for a mean number of vicariant events (Ψ) of one (*i.e.,* simultaneous divergence), with ∼20.1% of the posterior probability on that value (Fig. 4). However, the data also showed a long, right-hand tail with consistent, relatively high support (∼5%) for all Ψ values greater than one –up to the maximum Ψ of 18 (Table S7). When additional data were added to form the 3-gene dataset (Fig. 4), support for one vicariance event increased to 80.4% of the total, with a corresponding decrease in support for the tail of the distribution (Table S7). However, because estimates of the number of divergence events (Ψ) do not take the temporal spacing of these events into account, it is an unreliable estimator of the number of true divergence events (Overcast, Bagley & Hickerson, 2017).

**Figure 5:**
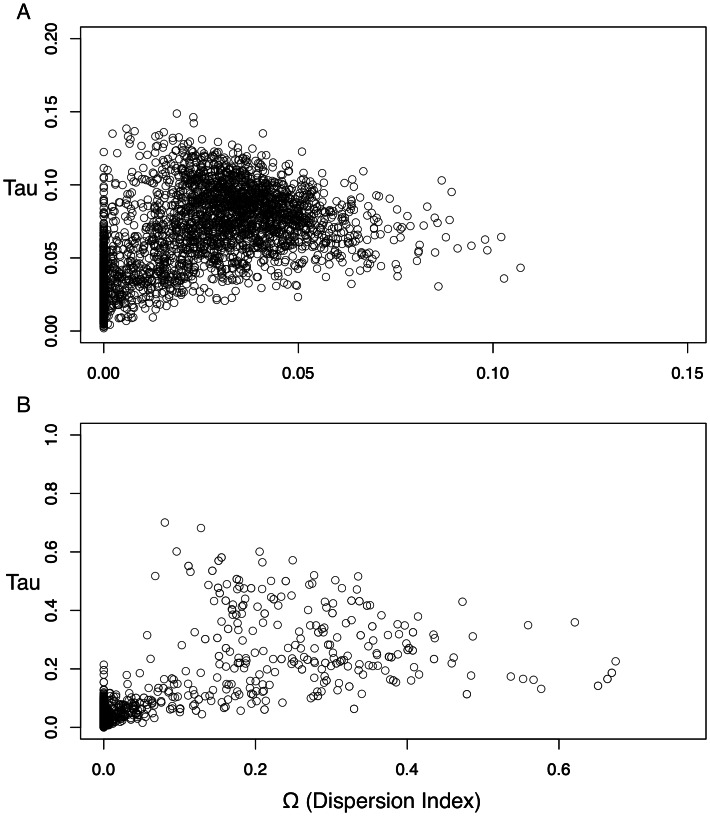
Scatterplots for dispersion index (Ω) values of the Ψ-unconstrained ND2 (A) and 3-gene (B) MTML-msBayes analyses. The bulk of the ND2 points cluster at values greater than zero, indicating strong support for nonsimultaneous divergence of taxa, despite a smaller clustering of points between zero and one. Conversely, the 3-gene values are drastically left-shifted, densely clustered on zero and produce an average Ω not significantly different from zero.

The dispersion index of divergence times (Ω), a direct test for the simultaneous or nonsimultaneous divergence of all comparisons, where nonsimultaneous divergence is indicated by a Ω significantly different from zero, also differed between these datasets. The ND2 dataset (Fig. 5) showed Ω values significantly different from zero (posterior mean of Ω = 0.0246, Bayes factor = 0 when comparing the probability distribution of Ω ≤ 0.01 and Ω > 0.01), indicating moderate support for more than one (*i.e.,* nonsimultaneous) vicariance event. The 3-gene dataset, in contrast, failed to produce a nonzero Ω-estimate, indicating strong support for simultaneous divergence.

## Discussion

We sought to determine how pulses of avian divergence events were distributed across Beringia, and how common these events were among lineages (*i.e.,* how many occurred overall, whether affecting one or more lineages). We began to answer these questions using pairwise comparisons of mitochondrial data from 39 closely related taxon pairs from 38 lineages across Beringia (Fig. 1), consisting of 432 individuals of 14 families (Table 1). The majority of events were found to occur in a clumped distribution across the nearest (most recent) half of the Pleistocene (Fig. 2). The strongest pulse occurs within the past 250,000 years, and comprises a substantial fraction of these comparisons, including all but two of the population-level comparisons. As the majority of these recently diverged lineages do not have significant *F*_*ST*_ values, this cohort may simply represent taxa that have not diverged yet or that have reticulated. The remaining lineages appear to have diverged continuously across the most recent half of the Pleistocene.

Both datasets statistically support simultaneous divergence for all taxa, and the larger, presumably less-biased 3-gene dataset supports it much more strongly (ND2 posterior probability = 20.1%, 3-gene posterior probability = 80.4%; Fig. 4). This is an unexpected and biologically implausible result, given the cyclic disturbance history of the system, the diverse set of avian lineages studied, and the widely varying, relatively continuous divergence date estimates among all pairwise contrasts. This is likely attributable to the statistical testing framework used. MTML-msBayes is designed to test for the simultaneous *vs.* nonsimultaneous divergence of lineages—*i.e.,* to test whether any two or more lineages shared the same vicariance event. The algorithm is agnostic to the density of lineages diverging within a given time period (Overcast, Bagley & Hickerson, 2017) and relies primarily on the presence of gaps in the dataset to demarcate vicariance events. As such, a sufficiently continuous spread of vicariance events might make it increasingly difficult to pick out distinct vicariance periods, and might, ultimately, become statistically indistinguishable from a single, strongly-supported divergence event. A buffer parameter was implemented in later versions of MTML-msBayes to attempt to alter this behavior, but this parameter has not been shown to be effective in improving the accuracy of estimations Overcast, Bagley & Hickerson, 2017; N Takebayashi and M Hickerson, pers. comm., 2022). Additionally, the mitochondrial genome is inherited uniparentally and does not recombine. The three genes we used are completely linked, and they do not give independent genealogical history, which is not consistent with the model. With this effectively single locus, the method might not give reliable results.

### Distribution of avian divergence events in Beringia

Our data indicate that, on average, avian lineages in Beringia differ in divergence depth based on taxonomic level, with species generally having diverged deeper in time than subspecies, and both, on average, having deeper divergences than populations (Fig. 2, Table 2). However, there is also substantial variation within categories, with taxonomic level explaining 45% of the variation in divergence timing. Other taxonomies are available in which different species limits are given than those used here for some taxa (*e.g.*, Withrow, Gibson & Winker, 2025; AviList Core Team, 2025). But these affect a minority of cases in our study and do not materially affect the unsurprising outcome that while mitochondrial divergence levels increase between taxonomic categories (Fig. 2, Table 2), they have considerable variation and overlap between categories and are not reliable indicators of the completion of speciation (cf., Winker, 2021). We make no taxonomic recommendations based on these results.

Our divergence date estimates were more recent than expected for lineages that had been previously studied (Table 1, Fig. 2; *e.g.*, Zink et al., 1995; Humphries & Winker, 2011). Divergence dates show some apparent clumping of events at around 1.3–1.8 Mya, but from 1.0 Mya toward the present divergences appear to be relatively continuous when all taxonomic levels are overlaid (Table 1, Fig. 2). The cluster of recent, population-level divergences from ∼0.0–0.3 Mya likely reflects a pulse of recent, and possibly ongoing divergences among these taxa, albeit with variable levels of gene flow across taxa. This is supported by pairwise *F*_*ST*_ values, which, with the exception of *Uria aalge*, were nonsignificant for all taxa in this cluster (Table 4). The two population-level comparisons outside of this cluster (*Aythya marila*, ∼629 Kya and *Uria lomvia*, ∼1 Mya; Table 1, Fig. 2) showed significant differentiation in *F*_*ST*_ for only one lineage (*A. marila*).

Divergence events here are strongly biased towards the more recent half of the Pleistocene, and none occur earlier than ∼1.9 Mya (Table 1, Fig. 2). The apparent lack of divergence events during the earliest third of the Pleistocene, followed by what seem to be one or a small number of events in the more recent two thirds, is unexpected. At face value, this result would suggest that avian speciation was only affected by (or at least, more strongly affected by) climatic cycles in the middle-to-late Pleistocene. Climate records (Ruddiman, Raymo & McIntyre, 1986) do not show marked changes in intensity or length of glacial cycles that would explain this apparent response. Also, prior research has suggested that divergences did occur in the early Pleistocene (Zink & Klicka, 2000; Lovette, 2005; Humphries & Winker, 2011; Winker et al., 2023).

One possible explanation for this apparent discrepancy is in the type of sequencing data used here to estimate divergence times *versus* the historic data and divergence calibrations using such data. The data in Humphries & Winker (2011) and Peters et al. (2014), both generated for similar taxa with equivalent markers, were developed using 1–2x Sanger sequencing, which generates a single consensus sequence from one or two sequencing reads, each deriving from many thousands of amplicons from one set of primers. Alternatively, Illumina sequences like those generated here, are the consensus of ∼100 individual reads, each derived from a single library molecule, which generates the cluster of sequenced molecules. Both methods can produce high-quality data, but the patterns and rates of errors are different (Fridman et al., 2021). High-coverage Illumina data can reduce the incidence of miscalled bases, reducing the frequency of errors in the data and, hence, decreasing overall mitochondrial divergence values *versus* data with more errors. This was empirically supported in a direct comparison between the prior Sanger and current, high-coverage Illumina ND2 data from the same specimens in the seven shared taxa between this study and that of Humphries & Winker (2011). Nucleotide diversity (*π*), used in this case as a rough proxy for error rate when considering the difference between two datasets, showed that divergence values calculated from Sanger sequence data were on average 1.67 times higher than values from the Illumina data (Table 5). Although per-sequence error rates are generally higher with Illumina instruments than traditional Sanger sequencing, our filter of discarding base calls with quality scores of <30, combined with our high coverage, creates datasets of higher quality than typical Sanger sequence data (Illumina, 2011). Variation in data quality among sequencing chemistries, instrumentation, and experiments is widely recognized (*e.g.*, Stoler & Nekrutenko, 2021), but we are unable to make direct comparisons between our data and corresponding historic Sanger datasets beyond pointing out that our filter produces data of nearly an order of magnitude higher quality than Sanger data (Q30 *vs.* ∼Q20; Illumina, 2011) and demonstrating higher variation in the latter (Table 5). Higher-quality data with fewer variants called likely had a major impact on our divergence date estimates (*e.g.*, Table 5).

**Table 5:** Comparison of ND2 nucleotide diversity (*π*) between sequencing technologies. Contrasts of nucleotide diversity (*π*) between low-coverage Sanger sequencing data (1–2×; from Humphries & Winker, 2011) and high-coverage Illumina data (∼90–495 ×) from this study for seven representative taxon pairs.

Taxon pair	Coverage (Sanger)	Coverage (Illumina)	Sanger nucleotide diversity (*π*)	Illumina nucleotide diversity (*π*)
Overall	1-2	270.7	0.025	0.015
*Anas c. crecca*/*A. c. carolinensis*	1-2	288.7	0.046	0.018
*Mareca penelope*/*M. americana*	1-2	270.6	0.012	0.01
*Clangula hyemalis* (Eurasia/N. American)	1-2	496.8	0.001	0.001
*Luscinia svecica* (Eurasia/N. America)	1-2	204.6	0.003	0.001
*Numenius phaeopus* (Eurasia/N. America)	1-2	213.4	0.024	0.019
*Pica pica*/*P. hudsonia*	1-2	89.6	0.037	0.018
*Pinicola enucleator kamtschatkensis*/*P. e. flammula*	1-2	378.6	0.039	0.026
*Tringa brevipes*/*T. incana*	1-2	222.9	0.04	0.028
Averages		270.7	0.025	0.015

Additionally, divergence time estimates here were likely impacted by the lack of an appropriate calibration, as the substitution rates from Lerner et al. (2011) were estimated from lower-coverage Roche 454 sequence data and would probably also result in an underestimate of divergence times when applied to data from high-coverage sequences (Luo et al., 2012). Thus, it is likely that the consistently earlier divergence dates generated in this study are an artifact of a mismatched high-coverage dataset (with its correspondingly lower variation than found in data from using older methods, *e.g.*, Table 5) and a calibration rate estimate based on lower-coverage data (Lerner et al., 2011), which would push our divergence estimates closer to the present.

Current methods of dealing with the problem of incorrectly calibrated avian molecular clocks, such as the calibration rates of Arcones, Ponti & Vieites (2021), are not applicable here. Arcones, Ponti & Vieites (2021) calibrated mtDNA divergence rates at the order level by using 622 full mitogenomes representing 33 extant families of birds, with fossil calibration points at depths of 11 to 53 Mya. These rates are suitable for estimating divergence times in deep evolutionary time within Aves, where saturation is high, reciprocal monophyly is assured, and the discrepancy between gene and population divergence is minimal (Arbogast et al., 2002). In our shallow-divergence dataset, Arcones, Ponti & Vieites’s (2021) rates produced divergence dates an order of magnitude older than previous analyses of Beringian birds (Maley & Winker, 2010; Humphries & Winker, 2011) and dates generated here using the more shallowly calibrated (1.9–5.7 Mya), passerine-specific rates estimated by Lerner et al. (2011; Table S6).

### Number of avian divergence events in Beringia

For the 18 taxa found to be significantly differentiated (Table 4), one vicariance event would represent complete synchrony and 18 would indicate complete asynchrony of divergence. Here, we find the most support for a single vicariance event, which is biologically surprising, given the staggered taxonomic depths and divergence times of these lineages. Dating calibration issues do not affect this outcome.

One explanation for this might be that successive cycles of isolation and gene flow, most likely driven by glacial-interglacial cycles, have muddied individual vicariance events, such that only the last one or two events are easily detectable. The exact number of vicariance events affecting these lineages is unclear due to the relative difficulty that MTML-msBayes has in estimating Ψ (number of divergence events) among variably temporally clustered divergence events (Overcast, Bagley & Hickerson, 2017). Thus, when considering the frequency of glacial-interglacial cycles (Lisiecki & Raymo, 2005) and the blurring effects on divergence that gene flow has likely had (Oliver Brown, Glenn & Winker, 2025), it might not be possible to sharpen our understanding of divergence clustering in this system.

## Conclusions

Avian divergence and speciation in Beringia appears to have occurred relatively continuously, but in one or perhaps more pulses across the most recent two thirds of the Pleistocene, with a small recent pulse of lineages close to the present representing lineages that may not have diverged at all yet. This is an unusual result given the cyclical nature of the most obvious abiotic driver for divergence in Beringia (glacial cycles) and might be due to multiple, effectively almost continuous vicariance events appearing statistically indistinguishable from a single, simultaneous vicariance event. Finally, it is also likely that the true divergence dates for each lineage studied here are older than reported, due to the lack of an appropriate dating calibration for relatively recent divergences and high-coverage, Illumina (or other similar low-error) sequence data.

##  Supplemental Information

10.7717/peerj.20675/supp-1 Supplemental Information 1 Supplementary Tables S1-S8 Supplementary Tables for Collier et al. “Mitogenomes reveal the timing and distribution of divergence events among trans-Beringian birds”.

10.7717/peerj.20675/supp-2 Supplemental Information 2 Estimated dates of Beringian divergence Date estimates are derived from ND2 JC-distance estimates, and the numbers by each point denote the taxon pair from Table S6. Black dots indicate the average value for each taxonomic level. Population-level comparisons are significantly lower than both species- and subspecies-level divergences, but species- and subspecies-level divergences are not different (Table 2).

10.7717/peerj.20675/supp-3 Supplemental Information 3 Code

10.7717/peerj.20675/supp-4 Supplemental Information 4 Raw genetic divergence values for all lineages All divergence metrics calculated in this study, for both the full mitogenome dataset and ND2 subset. Both JC-distance and net nucleotide divergence values are included.

10.7717/peerj.20675/supp-5 Supplemental Information 5 Raw mitochondrial divergence rates Divergence rates for multiple mitochondrial markers and avian orders, referenced from (Lerner et al., 2011; Arcones, Ponti & Vieites, 2021). These data were used to calculate full mitogenome divergence rates, and to scale Passeriform-specific full mitogenome and ND2 rates to multiple avian orders.

## Additional Information and Declarations

### Data Availability

The following information was supplied regarding data availability: The code and data are available in the Supplemental Files. The raw sequence data are available at NCBI BioProject: OR242768–OR243195.
