# Supplementary material for: Mitogenomes reveal the timing and distribution of divergence events among trans-Beringian birds
Source: PeerJ. 2026 Feb 2;14:e20675. doi: 10.7717/peerj.20675 (PMC12875246; doi:10.7717/peerj.20675)

Figure S1 Estimated dates of Beringian divergence

Date estimates are derived from ND2 JC-distance estimates, and the numbers by each point denote the taxon pair from Table S6. Black dots indicate the average value for each taxonomic level. Population-level comparisons are significantly lower than both species- and subspecies-level divergences, but species- and subspecies-level divergences are not different (Table 2).

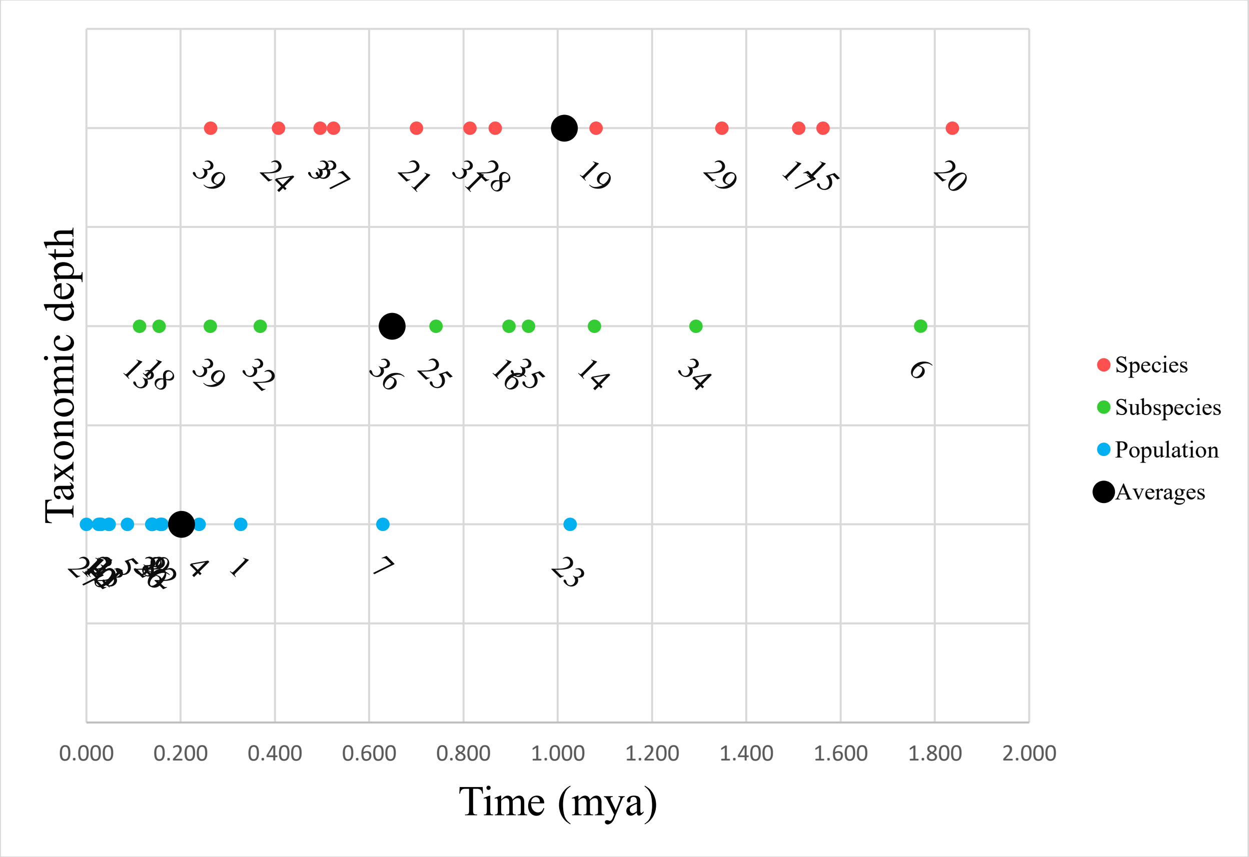

Supplement: Supplemental Information 2 — Date estimates are derived from ND2 JC-distance estimates, and the numbers by each point denote the taxon pair from Table S6. Black dots indicate the average value for each taxonomic level. Population-level comparisons are significantly lower than both species- and subspecies-level divergences, but species- and subspecies-level divergences are not different (Table 2). [file peerj-14-20675-s002.pdf]
